# Efficacy of subcutaneous tocilizumab in patients with rheumatoid arthritis and systemic sclerosis overlap syndrome: a report of two cases and review of the literature

**DOI:** 10.1186/s41927-019-0063-x

**Published:** 2019-04-24

**Authors:** Hiroki Wakabayashi, Hitoshi Kino, Makoto Kondo, Keiichi Yamanaka, Masahiro Hasegawa, Akihiro Sudo

**Affiliations:** 1 Department of Orthopaedic Surgery, Mie University Graduate School of Medicine, 2-174 Edobashi, Tsu, Mie 514-8507 Japan; 2 Kino Orthopedic Clinic, 4-1-7 Kikyogaoka, Nabari, Mie 518-0624 Japan; 3 Department of Dermatology, Mie University Graduate School of Medicine, 2-174 Edobashi, Tsu, Mie 514-8507 Japan

**Keywords:** Rheumatoid arthritis, Systemic sclerosis, Interleukin-6, Tocilizumab

## Abstract

**Background:**

The details of two rheumatoid arthritis (RA) patients with systemic sclerosis (SSc) who were administered tocilizumab, an anti-interleukin-6 receptor antibody, are reported, along with a review of the literature.

**Case presentation:**

Two RA patients with SSc with inadequate responses to disease-modifying antirheumatic drugs (DMARDs) were given tocilizumab 162 mg every 2 weeks for 18 months. RA disease activity was evaluated by the 28-joint disease activity score with erythrocyte sedimentation rate (DAS28-ESR) and the clinical disease activity index (CDAI). The skin condition of SSc was evaluated by pinching the skin according to the modified Rodnan total skin thickness score (mRSS). Softening of the skin and improvements of arthritis and the patient global assessment were observed during tocilizumab treatment, with reduction of not only RA disease activity, but also of the mRSS.

**Conclusion:**

Tocilizumab may be effective in patients with RA and SSc overlap syndrome for which conventional treatment is inadequate. Further research is needed because this report included only two patients.

## Background

Systemic sclerosis (SSc) is a connective tissue disease that develops sclerotic changes characterized by obliterative and proliferative microvascular involvement, activation of the immune system and increase of extracellular matrix deposition in the skin and various internal organs [1]. SSc presents stiffness of extremities due to sclerosis and joint swelling in the skin and periarticular connective tissues. Visceral involvement during the course of the disease (pulmonary, cardiac, gastrointestinal, and renal complications) is factors related to mortality [2, 3].

Interleukin 6 (IL-6) is a pleiotropic proinflammatory multifunctional cytokine such as T cell activation, initiation of acute-phase reactants (e.g., C-reactive protein), and stimulation of hematopoietic precursor cell growth, causing maturation of B cells into antibody-producing cells and cell differentiation. IL-6 overexpression and pathogenicity have been demonstrated in SSc as well as rheumatoid arthritis (RA) [4]. Tocilizumab (TCZ), an anti-IL-6 receptor antibody, blocks the functions of IL-6, and its efficacy for the treatment of RA, juvenile idiopathic arthritis, and Castleman’s disease has been verified [5].

A mini-series of two RA patients with refractory SSc (cases 1 and 2), treated with TCZ (162 mg every 2 weeks), is reported.

## Case presentations

### Case 1: a 74-year-old Japanese woman at the time of starting tocilizumab treatment

The patient had joint pain and swelling in 2003. She was diagnosed with RA and treated with disease-modifying antirheumatic drugs (DMARDs). In 2015, the right wrist joint was swollen and tender, and the CRP level increased to 2.83 mg/dl (normal value < 0.3 mg/dl). Although she received treatment for RA with methotrexate (MTX), salazosulfapyridine (SASP), and steroids, her arthritis was poorly controlled. In December 2015, because her arthritis worsened, she visited our hospital. Scleroderma from the fingers to the forearms was also observed at the first visit.

Anti-nuclear antibody (ANA) was 1280× (centromere), anti-cyclic citrullinated peptide antibody (ACPA) was 150 U/ml (normal value ≤4.5 U/ml), rheumatoid factor (RF) was 52 IU/ml (normal value ≤15 IU/ml), and anti-centromere antibody was 17.8 IU/ml (normal value ≤7.0 U/ml); all of them were elevated, but antibodies against topoisomerase I and U1-RNP were negative. The skin sclerosis developed from her fingers and expanded to her forearms, face, and feet. Chest computed tomography (CT) showed slight interstitial lung disease in the bilateral lower lung areas. The patient met the classification criteria for SSc established by the ACR/EULAR criteria in 2013 [6]. The patient was diagnosed with overlap syndrome involving RA and SSc. Larsen grade 3 was observed on both wrist and ankle X-rays, and grade 4 was observed on the left elbow X-ray. The 28-joint disease activity score with erythrocyte sedimentation rate (DAS28-ESR) and the clinical disease activity index (CDAI) were high, at 5.66 and 31.8, respectively. The modified Rodnan total skin thickness score (mRSS) was 23.

**Fig. 1:**
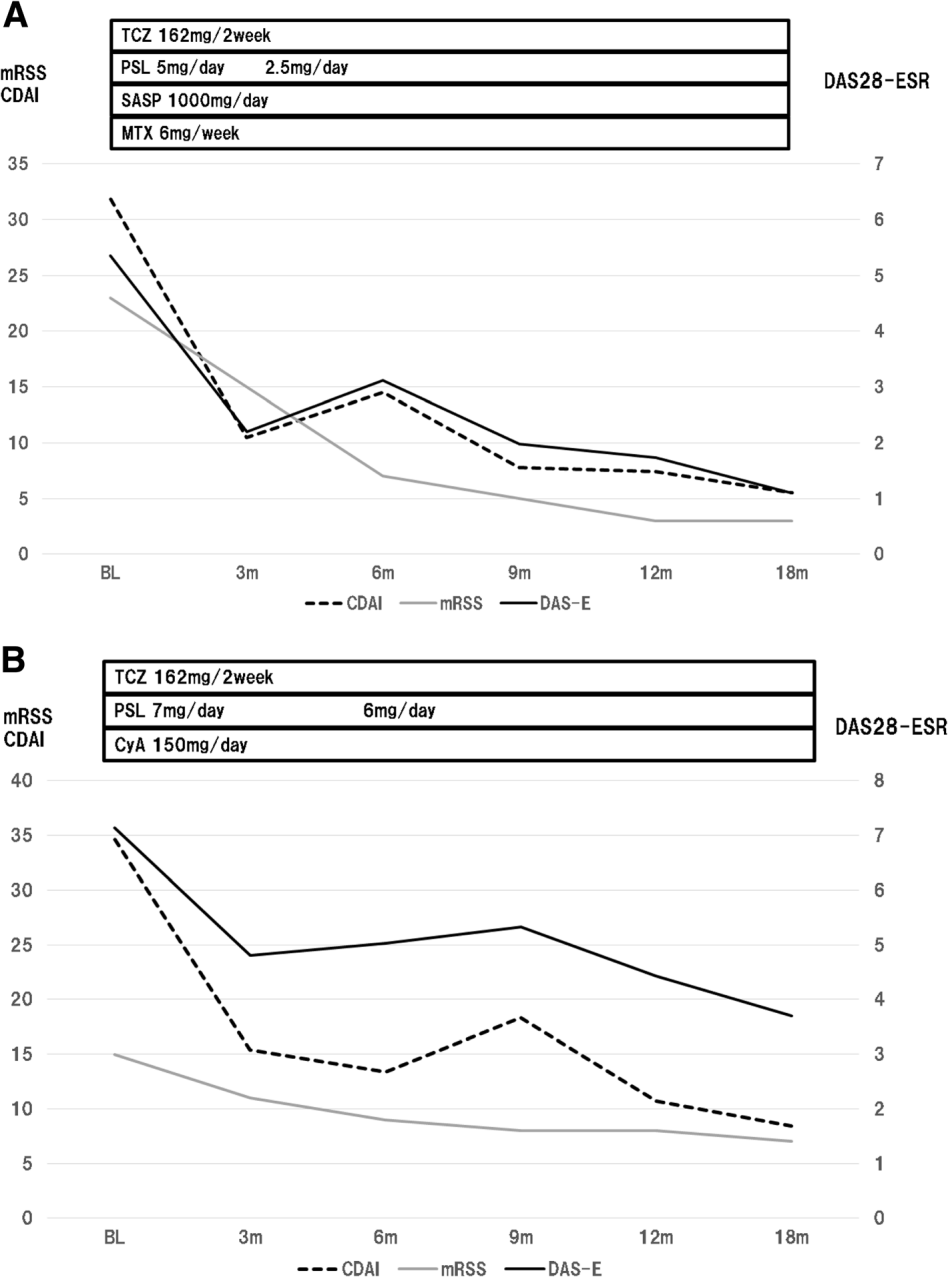
Clinical courses of case 1 (**a**) and case 2 (**b**) during tocilizumab therapy

Both the RA and SSc were judged to be active, and it was decided to treat the patient with TCZ, 162 mg every 2 weeks. Administration of steroid (prednisolone 5 mg/day) and DMARDs (MTX 6 mg/week and SASP 1000 mg/day) was continued (Fig. 1a). During the 18-month period of TCZ therapy, TCZ was well tolerated, and the patient experienced general improvement in normal daily activities. At 18 months, the patient global assessment improved by 71 (75 to 4), the DAS28-ESR decreased from 5.66 to 1.10, the CDAI decreased from 31.8 to 5.5, and skin thickness evaluated with the mRSS improved from 23 to 3 (Fig. 1a). Reductions of both RA disease activity and of mRSS were seen.

There were no remarkable adverse events. Interstitial pneumonia did not change during the treatment period.

### Case 2: a 65-year-old Japanese woman at the time of starting tocilizumab treatment

This patient with a history of RA diagnosed at 27 years of age underwent right wrist arthroplasty. After arthroplasty, she had been receiving sodium gold thiomalate (Shiosol®) for 7 years, which relieved her joint symptoms. However, she abruptly stopped outpatient visits, and was lost to follow-up.

In 2010, she was affected by joint pain, swelling and scleroderma of the palms, and visited our hospital again. The skin sclerosis developed from her fingers and expanded to her forearms, face, and feet. The findings of her right forearm skin biopsy were found to be consistent with SSc. She was diagnosed with overlap syndrome involving RA and SSc. She received treatment with cyclosporine, SASP, and steroids for RA and SSc treatment. Though an initial response was seen, the arthritis worsened. In 2016, both wrist joints were swollen and tender, and the CRP level increased to 1.05 mg/dl (normal value < 0.3 mg/dl). Larsen grade 3 was observed on wrist X-rays. A CT study showed patchy infiltrates associated with ground-glass opacities in the bilateral lower lung areas. ANA was 320× (homogeneous), ACPA was 151 U/ml (normal value ≤4.5 U/ml), RF was 229 IU/ml (normal value ≤15 IU/ml), and anti-topoisomerase I antibody was 88.7 U/ml (normal value ≤7.0 U/ml); all of them were increased, but antibodies against centromere and U1-RNP were negative. DAS28-ESR and CDAI were high, at 7.14 and 34.6, respectively. Skin sclerosis developed from her fingers to her forearms and face, and the mRSS was 15.

Both the RA and SSc were judged to be active, and it was decided to treat the patient with TCZ, 162 mg every 2 weeks. Administration of steroid (7 mg/day) and a DMARD (cyclosporine 150 mg/day) was continued (Fig. 1b). During the 18-month period of TCZ therapy, both RA disease activity and mRSS decreased. At 18 months, the patient global assessment improved by 32 (68 to 36), RA disease activity decreased (DAS28-ESR decreased from 7.14 to 3.70; CDAI decreased from 34.6 to 8.4), and skin thickness evaluated with the mRSS improved from 15 to 7 (Fig. 1b).

Interstitial pneumonia did not change during the treatment period. This patient developed cellulitis in the right foot plantar region at 6 weeks of treatment as an adverse reaction. TCZ was withdrawn for 4 weeks, but after the cellulitis resolved, she continued TCZ treatment.

## Discussion and conclusions

Recent progress in medicine has dramatically improved the treatment and mortality of patients with rheumatic diseases such as RA [7]. However, radical therapy for SSc has not been developed and remains disappointing, with high mortality.

IL-6 is a multifunctional cytokine that regulates immune responses and induces acute phase responses. Despite the critical physiological activities of IL-6, excessive production of IL-6 is pathologically involved in various immune-mediated inflammatory diseases, including RA [8].

Extensive studies have demonstrated that IL-6 plays a pivotal role in the pathogenesis of SSc. According to a previous report by Khan et al., IL-6 is upregulated in dermal fibroblasts, endothelial cells, and perivascular inflammatory cells in the majority of early diffuse cutaneous SSc cases [9]. Furthermore, high serum IL-6 levels are associated with the severity of skin sclerosis, as well as reduced survival [4, 9].

TCZ administration proved beneficial for the skin sclerosis in the present cases, as reported previously (Table 1) [10–17]. In a recent phase 2 trial, TCZ was shown to have promising effects on skin in SSc [14].

**Table 1:** Previous reports of treatment with tocilizumab in systemic sclerosis patients with or without rheumatoid arthritis

Author	Shima Y,	Fernandes das NM	Frech TM	Shima Y	Khanna D	Elhai M	Saito E	Kono M	Presentcases
Reference (Year)	Ref10 (2010)	Ref11 (2015)	Ref12 (2015)	Ref13 (2015)	Ref14 (2016)	Ref15 (2013)	ref16 (2014)	ref17 (2017)	2018
Diagnosis	SSc	SSc, (P3 SSc/RA)	SSc	SSc	SSc	SSc/Arthritis	SSc/RA	SSc/RA	SSc/RA
Country (centers)	Japan	Portugal	USA	Japan	USA	EULAR	Japan	Japan	Japan
Number of patients (n)	2	3	2	1	43	15	1	2	2
Age, years (SD or range)	42, 57	55, 42, 54	56, 68	59	51 ± 11.7	56 (45–61)	57	25, 32	74, 65
Duration of SSc, years (SD or range)	2, 3	3, 8, 1	ns, 0.25	4	1.46 ± 1.16	5 (4–9)	2.5	2, 3	1, 6
Durationof RA, years							2.5	0.25, 3	12, 38
Female, n (%)	1 (50%)	3 (100%)	2 (100%)	1 (100%)	32 (74%)	13 (86.7%)	1 (100%)	1 (50%)	2 (100%)
Anti-nuclear antibody	2 (100%)	3 (100%)	2 (100%)	+			+	2 (100%)	2 (100%)
Anti-RNA polymerase antibody			P1+, P2-	+	13 (30%)				
Anti-topoisomerase antibody	P1-, P2+	P1-, P2+, P3+		–	18 (42%)	10 (76.9%)		P1+, P2- P2:U1-RNP+	P1-, P2+
Anti-centromere antibody				–		1 (7.7%)			P1+, P2-
Anti-CCP antibody		P1-, P2-, P3+				3 (37.5%)	+	2 (100%)	2 (100%)
Rheumatoid factor		P1-, P2-, P3+				3 (37.5%)	+		2 (100%)
Previous biological drugs		P3: ADA, ETN				RTX:3, ABT:1, TNF: 2	IFX		
Immunosuppressive drugs	P1: CyA	P1: CyA,AZA,HCQ P2: CyA,AZA P3: MTX,HCQ			22 (51%)	MTX: 8 (57.1%)	MTX, TAC, SASP	P1: IVCY,TAC P2: SASP	P1: MTX, SASP P2: CyA
Concomitant systemic corticosteroid use	2 (100%)	3 (100%)		+	21 (49%)	11 pts. (73.3%) [≤10 mg]		2 (100%)	2 (100%)
Follow-up (months)	6	6	several, 26	16	12	5 (3 to 11.5)	9	12	18
Tocilizumab dose	8 mg/kg/4 w iv	8 mg/kg/4 w iv	ns	8 mg/kg/4 w iv	162 mg/w	8 mg/kg/month iv	600 mg/month	P1: iv, P2: sc	162 mg/2 w
mRSS baseline	27, 26	17, 41, 7	22, 27	35	26.4 ± 7.2	15 (4.5 to 24.0)		25, 14	23, 15
mRSS last infusion	13, 20	11, 25, 5	17, 6	7	19.6 ± 10.1	12 (3.8 to 16.3)	Not improved	8, 5	3, 7
DAS baseline		P3: 3.82				5.2 (3.9 to 6.1)	5.39	2.92, 6.92	5.66, 7.14
DAS last infusion		P3: 2.87				2.8 (2.2 to 3.4)	1.53	1.76, 1.1	1.10, 3.70
PGA baseline		70, 70, 60							75, 68
PGA last infusion		40, 30, 10							4, 36
Discontinued for lack of efficacy					1 (2.3%)	2 (13.3%)			
Discontinued for adverse events			1(gastroenterology)		5 (11.6%)				

*P1–3* patients 1–3, *iv* intravenous, *sc* subcutaneous, *ns* not shown

*CyA* cyclosporine, *AZA* azathioprine, *HCQ* hydroxychloroquine, *MTX* methotrexate, *IVCY* intravenous cyclophosphamide, *TAC* tacrolimus, *ADA* adalimumab, *ETN* etanercept, *IFX* infliximab, *TNF* tumor necrosis factor, *RTX* rituximab, *ABT* abatacept, *DAS* disease activity score, *PGA* patient global assessment

Although there have been many cases in which TCZ improved sclerosis of the skin, in some patients, TCZ was stopped for inefficacy with respect to skin sclerosis and/or adverse events. Elhai et al. reported that, in two patients (13.3%) [15], TCZ was stopped because of inefficacy after 3 months. The faSScinate study, a phase II trial, reported that treatment was discontinued because of inefficacy in one patient (2.3%) [14]. Since the stage of the patient’s skin thickening was late phase and involved only the fingers, it might be difficult to evaluate the improvement of skin thickening [16]. In the present study, the reason why skin improvement was small in case 2 may be the longer disease duration of SSc (Table 1).

The most common adverse events in the faSScinate study were infections, gastrointestinal disorders, skin or subcutaneous disorders, and musculoskeletal or connective tissue disorders. Treatment was withdrawn for adverse events in 11.6% (5 patients) of patients after 12 months. Although case 2 in the present study had cellulitis of the right foot plantar region, she continued TCZ after the cellulitis resolved.

In the present study, two patients with overlapping RA and SSc who were successfully treated with TCZ for polyarthritis as well as skin sclerosis were reported. TCZ administration clearly resulted in improvement of joint disease activity and skin scores in these cases, as well as previously reported cases of RA and SSc overlap patients. TCZ treatment may be useful in patients with RA and SSc overlap syndrome for whom conventional treatment is inadequate. However, as a limitation, the current report included only two cases, and it is therefore difficult to draw any conclusions. Further research is needed.

## Abbreviations

ACPA: Anti-cyclic citrullinated peptide antibody
ANA: Anti-nuclear antibody
CDAI: Clinical disease activity index
CRP: C-reactive protein
CT: Computed tomography
DAS: Disease activity score
DMARDs: Disease-modifying anti-rheumatic drugs
ESR: Erythrocyte sedimentation rate
IL-6: Interleukin-6
mRSS: modified Rodnan total skin score
MTX: Methotrexate
RA: Rheumatoid arthritis
RF: Rheumatoid factor
RNP: Ribonucleoprotein
SASP: Salazosulfapyridine
SSc: Systemic sclerosis
TCZ: Tocilizumab
